# First person – Leon Green

**DOI:** 10.1242/bio.043141

**Published:** 2019-03-15

## Abstract

First Person is a series of interviews with the first authors of a selection of papers published in Biology Open, helping early-career researchers promote themselves alongside their papers. Leon Green is first author on ‘Sperm-duct gland content increases sperm velocity in the sand goby’, published in BIO. Leon is a PhD student in the lab of Charlotta Kvarnemo at the University of Gothenburg, Sweden, investigating how fish adapt to the abiotic environment.


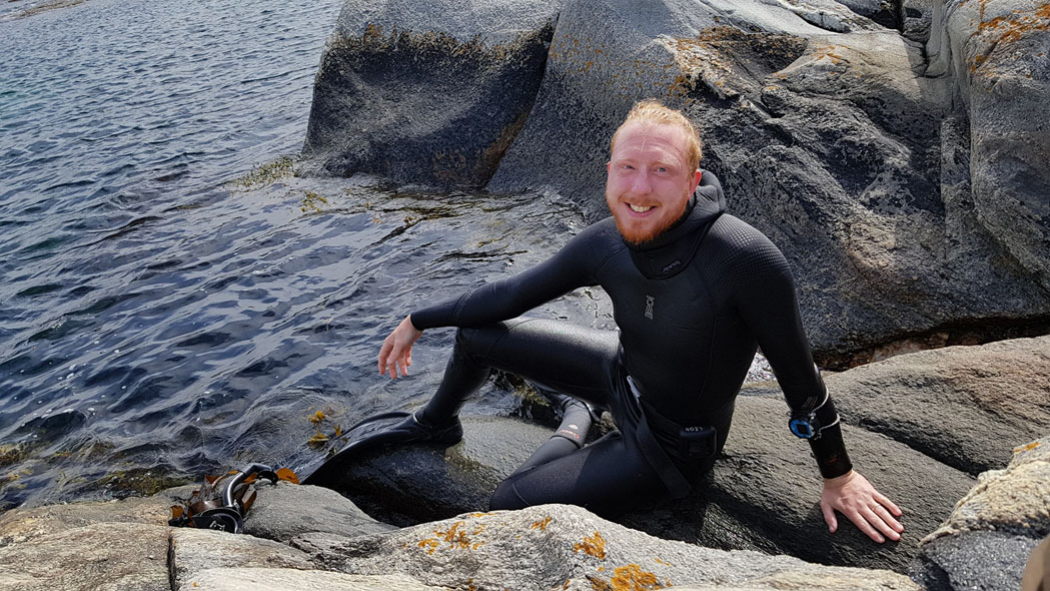


**Leon Green**

**What is your scientific background and the general focus of your lab?**

My background is in animal ecophysiology where I studied everything fishy, from sharks in the Atlantic to coral-reef fish in the Pacific Ocean. After my master’s thesis at the University of Gothenburg, I worked as a lab technician employed in various research projects associated with fish physiology and reproduction. I now study adaptations to the abiotic environment in traits associated with reproduction, mainly in fishes with paternal care, under the tutelage of Professor Charlotta Kvarnemo. My studies have taken a turn into evolutionary ecology, and I love mixing mechanistic thinking with more ultimate research questions in evolution.

“My studies have taken a turn into evolutionary ecology, and I love mixing mechanistic thinking with more ultimate research questions in evolution.”

**How would you explain the main findings of your paper to non-scientific family and friends?**

There are many ways to ‘boost’ your sperm if you are a male. These fishes we study, the sand gobies, do this through chemical substances produced in a specialized tissue next to the testis.

**What are the potential implications of these results for your field of research?**

Our findings will help us understand how these adaptations are spread across this family of fishes, and whether or not they work the same way in all of them.

**What has surprised you the most while conducting your research?**

That the substances produced by these glands didn't affect sperm survival over time. Increased velocity usually has consequences for viability, but we couldn't find such an effect in our experiments.

**What, in your opinion, are some of the greatest achievements in your field and how has this influenced your research?**

The greatest achievement in the field of reproductive biology are the findings regarding how complex the reproductive systems of vertebrates really are. There are so many steps, all the way from courting between individuals down to what chemicals help modulate how the sperm finds the egg, and even further on to how the parents invest in their offspring during their growth and development. No one can investigate everything at once, so we all do our own small contributions to the greater picture. In our case, it is finding out what these specialized glands really do for these fishes’ spermatozoa.

“No one can investigate everything at once, so we all do our own small contributions to the greater picture.”

**What changes do you think could improve the professional lives of early-career scientists?**

I believe early-career scientists need freedom enough to develop their own ideas, but also knowledge enough to put them into context. As a senior scientist, a forgiving attitude towards your protégés is key. Expectations should be on scientific soundness and robustness, rather than on making the next big finding in your scientific field. Luckily for me, I'm in a good spot.

**What's next for you?**

I will continue to study reproductive adaptations, but in invasive fishes and in a more direct environmental context. Gobies are some of the most invasive fishes on the planet and they have huge environmental impacts. Understanding where and how they can reproduce is important to mitigate their spread.

**Why study sperm?**

Sperm and eggs are really just extensions of our own phenotype! We think of them as the next life stage, as not being of ourselves. But if you look at it closely, they are just a continued part of our own phenotype, no less a part of ourselves than the cells in any other part of our body. This makes all of us organisms with a common ancestor just one big, continued and extended phenotype that stretches across the globe and across time. The thought makes you dizzy!

**Figure:**
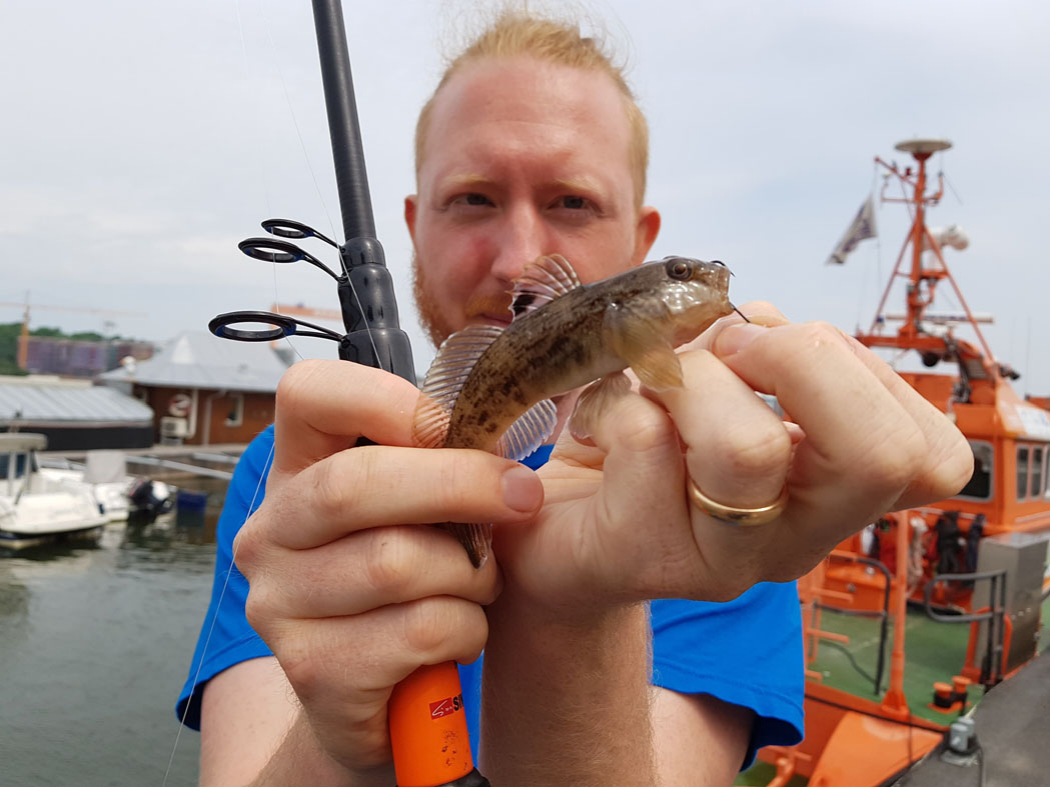
**Leon Green captures invasive gobies (*Neogobius melanostomus*) in the harbour of Gothenburg, Sweden.**
